# Case–control study on TP73 rs1801173 C > T gene polymorphism and susceptibility to gastric cancer in a Chinese Han population

**DOI:** 10.1186/s12920-021-01151-2

**Published:** 2022-01-24

**Authors:** Huiwen Pan, Xuyu Gu, Xiaoyan Wang, Zhenjun Gao, Guowen Ding, Chen Zou, Yu Fan

**Affiliations:** 1 Cancer Institute, the Affiliated People’s Hospital of Jiangsu University, Jiangsu, China; 2 Digestive Department, the Affiliated Suqian First People’s Hospital of Nanjing Medical University, Nanjing, China; 3 Digestive Department, Qingpu Branch Hospital of Affiliated Zhongshan Hospital, Fudan University, Shanghai, China

**Keywords:** TP73 gene, Gastric cancer, Single nucleotide polymorphism (SNP), rs1801173

## Abstract

**Background:**

This study investigated the role of TP73 gene polymorphism, rs1801173on risk of gastric cancer.

**Methods:**

We conducted a case-controlled study including 577 primary gastric cancer and 678 normal control cases. The target gene fragment was amplified using PCR using blood samples collected from patients. Allele analysis and genotyping were performed using snapshot method.

**Results:**

The findings showed that the control group had consistent genotype frequency distribution and presented Hardy–Weinberg equilibrium. The results showed no significant differences in sex, drinking history and age distributions between subjects with the polymorphism and subjects in the control group. Smoking status was correlated with incidence of gastric cancer (*P* = 0.006). The rs1801173 locus of TP73 gene contained 3 genotypes including: TT, CT, and CT. Logistic regression analysis showed that distribution of recessive model and dominant model was comparable between the two groups before (*P* = 0.688; 0.937) or after (*P* = 0.703; 0.990) adjusting for confounders. The distribution frequency in case group was not significantly different relative to that of the control group (*P* = 0.763).

**Conclusion:**

Smoking can independently influence the risk of gastric cancer. TP73 gene rs1801173 polymorphism was not significantly correlated with risk of gastric cancer.

**Supplementary Information:**

The online version contains supplementary material available at 10.1186/s12920-021-01151-2.

## Background

Gastric cancer (GC) is a malignant tumor characterized by high incidence worldwide. It is the third leading cause of cancer-related deaths, despite a recent decline in overall incidence[1]. Each year, approximately 100,000 new cases of GC are reported, with GC-related mortality exceeding 700,000[2]. Approximately 50% of patients with GC present with metastases during diagnosis[3]. The progression landscape of gastric cancer is complex and involves numerous factors. Genetic factors such as single nucleotide polymorphisms (SNPs) are implicated in GC development in addition to environmental risk factors including helicobacter pylori (HP) infection and diet. SNPs, or polymorphisms are mutations that affect single nucleotides of genomes. SNPs are the most frequent forms of genetic variations in humans, representing more than 90% of all known morphology[4]. The tumor protein P73 (TP73) belongs to TP53 family. This family includes well-defined tumor suppressors, TP53 (p53) and TP63 (p63). These family members have a wide range of functions, including differentiation, tumor suppression, reproduction, aging, genome repair, stem cell biology, changes in epigenetic markers, metabolic processes, and embryonic development [5–8]. Compared with the frequently mutated TP53 gene, TP73 has no reported mutations[9]. G4A (rs2273953) and C14T (rs1801173) are key SNPS located on exon 2 of TP73 gene. These two SNPs are completely in a state of linkage disequilibrium with each other, hence denoted by G4C14-A4T14[10, 11]. Although TP73 gene is implicated in development of cancer, cancer epidemic patterns associated with TP73 polymorphisms (G4C14-A4T14) have not been fully elucidated. In previous research reports, TP73 G4C14-A4T14 polymorphism has been associated with the occurrence of numerous cancers [12–15]. On the other hand, we did not focus exclusively on TP73 rs1801173 loci in our overall study, but we selected 20 genes and 90 SNPs potential related genes and loci as overall research. Our three articles were published (PMID: 33363398; 32655638; 32753933), while this article mainly introduces TP73 rs1801173 loci. As such, the current study is part of a larger body of work referencing our previous studies. This study adopted a case–control design to explore alleles and TP73 gene rs1801173 genotypes in GC patients and explore the role of TP73 genetic status and on risk of gastric cancer. Patient characteristics including drinking history, smoking history, age, sex, were recorded. The relationship between these factors and TP73 SNPs was evaluated to lay a foundation for timely diagnosis and effect management of gastric cancer.

## Methods

### Study population and method

The present study was conducted following a procedure described previously[16]. Study population: 678 healthy subjects and 577 consecutive GC patients were recruited from Affiliated People’s Hospital of Jiangsu University from May 2013 to June 2017. Ethics statement: The Ethics Committee of Affiliated People’s Hospital of Jiangsu University provided an approval for the current study. Patients and controls provided written informed consent. Questionnaires were used to obtain data on patient characteristics and clinical characteristics were retrieved from the hospital medical records. Extraction of DNA and genotype analysis: Peripheral blood from each subject was used for DNA extraction. ExoI and FastAP were used to purify PCR amplicons and the further analysis conducted to extend the products. ABI3730XL was utilized to analyze the sequence for determination of the genotypes. Snapshot method was used to explore polymorphism, and the results were validated using 5% of the samples. The raw data named Additional file 1 that support the present findings was included in the supplementary information file.

GC group samples were selected using random sampling method. Sample power software was used for sample quantification. The power of test statistic was set at 80%, approximately 8% or more was used as the variation genotype frequency, the Minor Allele Frequency (MAF) was set above 5%, and the two-sided test with α = 0.05 as the significance level was used. The odds ratio (OR) was approximately 1.23/0.81 as indicated by the Power and Sample Size Calculation software (Power and Sample Size Calculations, Version 3.0, January 2009). The size of randomly selected GC sample in the present study met the criteria for genotypic studies.

Effects of patient characteristics on genotypes were explored using logistic regression analysis. Key performance indicator was evaluated. The relationship between TP73 and patient characteristics was explored using multivariate and univariate analyses. Multivariate analysis was used to explore whether alcohol consumption, sex, smoking and age were independent variables.

Models: CC represents the wild type, CT represents the heterozygous mutant genotype, and TT represents the homozygous mutant genotype.

Recessive model: TT versus (CT + CC); Dominant model:(CT + TT) versus CC; Additive model: TT versus CC; Super-dominant model: (CC + TT) versus CT.

### Snapshot method

The technology, developed by Applied Biology, Inc. (ABI), uses the principle of fluorescent-labeled single base extension typing, also referred to as mini-sequencing, and is intended for use in medium throughput SNP typing projects. In this method, SNPs at predetermined locations are explored using single tube reaction. An unlabeled oligonucleotide primer (or primers) is subjected to dideoxy single-base extension. DNA polymerase and fluorescently labeled ddNTPs are added and the primers bind to a complementary sequence. The primer is extended by one nucleotide at a time through, addition of a single ddNTP to the 3´ end of the primer by DNA polymerase. Added bases are identified through fluorescence analysis.

### Statistical analysis

Data analysis was carried out using SPSS version 20.0 (SPSS Inc., Chicago, IL, USA). Identified polymorphism distributions were analyzed using Chi-square test to see if they meet Hardy–Weinberg equilibrium requirements. The relationship between SNP alleles and genotypes and risk of GC was explored using logistic regression analysis.

## Results

The findings indicated that rs1801173 of TP73 gene was present on the first chromosome (Table 1). It plays a role in coding of proteins. In our controls, minor allele frequency (MAF) of rs1801173 is 0.267. Our controls have a Hardy–Weinberg equilibrium value of 0.229 (*P* > 0.05). This implies highly representative sample population was used in this study. 98.96% successful tests were obtained using the snapshot method.

**Table 1:** Primary information for gene TP73 gene rs1801173 polymorphisms

Genotyped SNPs	Gene	Chr Pos (NCBI Build 38)	Category	MAF for Chinese in database	MAF in controls	*P* value for HWE test in controls	Genotyping method	Genotyping value (%)
rs1801173	TP73	1:3682346	5_prime_UTR_variant, genic upstream transcript variant	0.267	0.229	0.821	Snapshot	98.96

MAF, minor allele frequency; HWE, Hardy–Weinberg equilibrium

The environmental risk factors and demographics of study subjects are presented in Table 2. The case group showed higher smoking rate relative to that of control subjects (34.49% vs. 27.29%, *P* = 0.006), age and sex were similar between the two groups (*P* = 0.635 and *P* = 0.698 respectively). This implies that gastric cancer pathogenesis and progression are not influenced by smoking status. Rate of alcohol consumption in GC patients was not significantly different compared with that of healthy subjects (*P* = 0.443).

**Table 2:** Distribution of selected demographic variables and risk factors in gastric cancer cases and control

	Overall cases (n = 577)	Overall controls (n = 678)	*P*
	n (%)	n (%)	
Age (years)	61.34 ± 11.097	62.31 ± 7.549	0.065
Age (years)			
< 62	268 (46.45)	324 (47.79)	
≥ 62	309(53.55)	354(52.21)	0.635
Sex			
Male	394 (68.28)	456(67.26)	
Female	183(31.72)	222(32.74)	0.698
Smoking status			
Never	378 (65.51)	493(72.71)	
Ever	199(34.49)	185 (27.29)	**0.006**
Alcohol use			
Never	453 (78.51)	520(76.70)	
Ever	124 (21.49)	158(23.30)	0.443

Bold value indicates statistically significant (*P* < 0.05)

**Table 3:** TP73 gene rs1801173 polymorphism in GC cases and controls and logistic regression analysis

Genotype	GC cases (n = 577)		Controls (n = 678)		Crude OR (95% CI)	*P*	Adjusted OR^a^ (95% CI)	*P*
	n	%	n	%				
rs1801173								
CC	341	60.67	396	59.55	1.00		1.00	
CT	190	33.81	233	35.04	0.95 (0.75–1.20)	0.657	0.95 (0.75–1.21)	0.691
TT	31	5.52	36	5.41	1.00 (0.61–1.65)	1.000	1.00 (0.77–1.28)	0.979
TC + TT	221	39.33	269	40.45	0.95 (0.76–1.20)	0.688	0.98 (0.81–1.00)	0.703
TT	31	5.52	36	5.41	0.98 (0.60–1.61)	0.937	1.00 (0.78–1.29)	0.990
CC + TC	531	94.48	629	94.59	1.00		1.00	

^a^ adjusted

Analysis of the distribution of rs1801173 SNP indicated that the distribution frequency of CT heterozygous mutations based on wild-type CC was comparable between the two groups (*P* = 0.657). In addition, alcohol consumption, smoking, age and sex did not vary after adjustment using logistic regression (*P* = 0.691). The distribution frequency of TT homozygous mutants in the two groups was similar before (*P* = 1.000) and after adjustment of confounding factors (*P* = 0.979). The dominant model showed significant difference in the frequency distribution of TC + TT mutations distribution in the two groups before (*P* = 0.688), and after adjustment of confounding factors (*P* = 0.703). Of note, the frequency distribution of distribution of TC + TT mutations as determined using the recessive-model group was not altered (*P* = 0.937). Drinking status, smoking status, age and sex were comparable between the two study groups (*P* = 0.990) (Tables 3, 4).

The findings showed that rs731173 allele frequency distribution of TP73 gene was not significantly different between case group and healthy group (*P* = 0.852, *P* = 0.619, and *P* = 0.917).

Table 5 tabulates rs1801173 polymorphism in TP73 gene according to stratification results: wild-type CC represents the reference genotype, TC indicates wild-type genotype, TT represents the homozygous genotype, dominant model, and recessive model, with no statistical significance in each group.

**Table 4:** Analysis of TP73 gene rs1801173 alleles between cases and controls

Locus	Variable	Case	Control	*P*	OR (95% CI)
rs1801173	C allele	872 (77.58)	1025 (77.07)		
	T allele	252 (22.42)	305 (22.93)	0.763	0.97 (0.80–1.17)

**Table 5:** Stratified analyses between TP73 gene rs1801173 polymorphism and risk by sex, age, smoking status and alcohol consumption

Variable	(Case/control)			Adjusted OR (95% CI); *P*				
	CC	TC	TT	CC	TC	TT	(TC + TT) versus CC	TT versus (CC + TC)
Sex								
Male	229/253	131/168	22/23	1.00	0.86 (0.65–1.15); *P*:0.314	1.06 (0.57–1.95); *P*:0.859	0.89 (0.67–1.17); *P*:0.389	0.89 (0.49–1.63); *P*:0.715
Female	112/143	59/65	9/13	1.00	1.16 (0.75–1.78); *P*:0.502	0.88 (0.37–2.14); *P*:0.785	1.11 (0.74–1.68); *P*:0.607	0.84 (0.35–2.02); *P*:0.700
Age								
< 62	163/182	90/117	8/16	1.00	0.86 (0.61–1.22); *P*:0.390	0.56 (0.23–1.34); *P*:0.186	0.82 (0.59–1.15); *P*:0.255	1.69 (0.71–4.02); *P*:0.228
≥ 62	178/214	100/116	23/20	1.00	1.04 (0.74–1.45); *P*:0.833	1.38 (0.74–2.60); *P*:0.313	1.09 (0.79–1.49); *P*:0.602	0.73 (0.39–1.36); *P*:0.324
Smoking status								
Never	228/290	119/172	21/24	1.00	0.87 (0.65–1.16); *P*:0.331	1.11 (0.60–2.05); *P*:0.731	0.91 (0.69–1.20); *P*:0.498	0.86 (0.47–1.57); *P*:0.619
Ever	113/106	71/61	10/12	1.00	1.09 (0.71–1.68); *P*:0.691	0.78 (0.32–1.89); *P*:0.583	1.04 (0.69–1.57); *P*:0.849	1.32 (0.56–3.14); *P*:0.526
Alcohol consumption								
Never	273/305	144/180	23/28	1.00	0.89 (0.68–1.18); *P*:0.420	0.68 (0.39–1.16); *P*:0.156	0.90 (0.69–1.17); *P*:0.414	1.42 (0.83–2.42); *P*:0.196
Ever	68/91	46/53	8/8	1.00	1.16 (0.70–1.92); *P*:0.561	1.34 (0.48–3.75); *P*:0.578	1.19 (0.73–1.92); *P*:0.491	0.79 (0.29–2.17); *P*:0.650

For TP73 gene rs1801173 the genotyping was successful 98.96% in 577 cases and 678 controls

Genotype analysis of TP73 gene rs1801173 was accurate (99.20%) in 678 controls and 577 cases.

## Discussion

Gastric cancer (GC) is caused by several factors including inflammation, infections, environmental factors, immune factors, genetic factors, and diet. SNPs regulate expression and function of genes. Studies on are important in elucidating pathogenesis of GC. P53 family comprises various genes including P73. P73 gene is found on chromosome lp36.33 in humans. The protein encoded by P73 is structurally and functionally similar to P53. P73 acts by transcriptionally activating p21WAFl/CIPI and other P53 target genes, inhibiting normal and transformed cell growth and promoting apoptosis [17]. These findings indicate that P73 gene is a potential anti-oncogene, implicated in tumor development and occurrence. Two SNP including G4A (rs2273953) and C14T (rs1801173) of TP73 at affect base 4 (G > A) and 14 (C > T), are in linkage disequilibrium with each other, hence referred to as G4C14-A4T14. G4C14-A4T14, located at the upstream of TP73 promoter in exon 2, can influence TP73 expression through a stem-loop structure. Recent studies indicate that p73 gene g4c14-a4t14 polymorphism is implicated in tumor susceptibility, subjected to racial and tumor type differences.

Studies by Yang et al. [18] and Niwa et al. [19] indicated that ehe two SNPs were not correlated with risk of cervical cancer in Uighurs and Japanese, respectively. However, Craveiro et al. [20] revealed that G4C14-A4T14 SNPs increases susceptibility to cervical cancer. Feng et al. [21] Hamajima et al. [22] reported the genotype frequencies of subjects with colorectal cancer and normal subjects were not significantly different. In contrast, findings of a in Korean population by Lee et al. [23] indicated that AT/AT genotype and GC/AT genotype were significantly correlated with colorectal cancer risk. Moreover, Arfaoui et al. [24] reported that genotype frequencies in cancer patients and health patients were significantly different. The findings indicated that the AT/AT genotype was associated with poor prognosis in patients with colorectal cancer. Other researchers studied the role of TP73 gene conducted in lung cancer. Hu et al. [25] reported that GC/AT and AT/AT genotypes were correlated with a remarkably decreased risk for lung cancer. Findings from a study by Li et al. [26] indicated that GC/AT AT/AT variants had a significant association with high risk for lung cancer. Choi et al. [27] contradicted both of them, demonstrating that TP73 G4C14-A4T14 polymorphism does not affect lung cancer susceptibility in Korean subjects. Zheng et al. [28] reported that p73 rs1801173 C > T SNP was linked with high risk of esophageal cancer [29]. However, studies have not explored the role of p73 gene polymorphism in susceptibility of gastric cancer. Studies should explore p73 rs1801173 C/T SNP to explore the relationship between the SNPs and risk of GC.

Based on the background above, this study examined rs1801173 locus polymorphism of TP73 gene in patients with gastric cancer cases and healthy subjects. The genotype frequency distribution at the site met criteria of Hardy–Weinberg equilibrium law, and the sample remained to have good population representativeness. This study found that the rate of smoking of case subjects was higher relative to the frequency of healthy controls (34.49% vs. 27.29%). The findings showed significant difference in distribution frequency of SNPs between the groups implying that smoking was associated with the occurrence and development of GC. The results showed that sex was an independent risk factor for GC. Men are more likely to develop gastric cancer than women, as determined by the random sampling method. This may relate to men who are more inclined to smoke and drink. Men are also significantly more likely to smoke and drink than women. Indeed, this is only speculation, and additional research and sample size verification are required. The alcoholic drinking status of the case group was not significantly different compared with that of the healthy controls. No statistical significance was observed for allele frequency of rs731173 locus in TP73 gene between GC patients and healthy subjects. Gene model and distribution of variants of GC patients was not significantly different compared with that of healthy controls. This finding cannot be used to infer the role of TP73 gene in GC. Moreover, alcohol consumption, smoking, and sex of different genotypes in rs1801173 locus of the case group were analyzed, demonstrating no significant association with susceptibility to gastric cancer. The negative results obtained in this study might be influenced by the following factors: presence of biases, insufficient genetic marker sites, and small sample size used in the study. As a result, the findings may have limitations that preclude conclusively ruling out an correlation between TP73 gene and gastric cancer. Moreover, TP73 SNPs is likely associated with environmental factors or other gene variants owing to the role of interactions between genes and environmental and genetic interactions in initiation and progression of several diseases, mainly chronic diseases. This interaction may modulate occurrence of GC. Further studies should explore gene-environment interactions and interactions between various genes to elucidate etiology of gastric cancer.

## Conclusions

Smoking is independently associated with occurrence of GC. TP73 gene rs1801173 polymorphism is not significantly correlated with susceptibility of gastric cancer.

## Supplementary Information

**Additional file 1**. Supplementary information file.

## Data Availability

The data that support the findings of the present are included in the supplementary information file named Additional file 1.

## Abbreviations

HP: Helicobacter pylori
GC: Gastric cancer
TP73: Tumor protein P73
PCR: Polymerase chain reaction
OR: Odds ratio
MAF: Minor Allele Frequency
SNPs: Single nucleotide polymorphisms

## References

[1] Siegel RL, Miller KD, Jemal A (2016). Cancer statistics, 2016. CA Cancer J Clin.

[2] Ohtsu A (2008). Chemotherapy for metastatic gastric cancer: past, present, and future. J Gastroenterol.

[3] Karadeniz M, Erdogan M, Berdeli A (2014). Association of interleukin-6 -174 G>C promoter polymorphism with increased risk of type 2 diabetes mellitus patients with diabetic nephropathy in Turkey. Genet Test Mol Biomark.

[4] Tozluoglu M, Karaca E, Haliloglu T (2008). Cataloging and organizing p73 interactions in cell cycle arrest and apoptosis. Nucl Acids Res.

[5] Tozluoglu M, Karaca E, Haliloglu T (2008). Cataloging and organizing p73 interactions in cell cycle arrest and apoptosis. Nucleic Acids Res.

[6] Jost CA, Marin MC, Kaelin WC (1997). p73 is a simian [correction of human] p53-related protein that can induce apoptosis. Nature.

[7] Costanzo A, Pediconi N, Narcisi A (2014). TP63 and TP73 in cancer, an unresolved “family” puzzle of complexity, redundancy and hierarchy. FEBS Lett.

[8] Candi E, Agostini M, Melino G (2014). How the TP 53 family proteins TP 63 and TP 73 contribute to tumorigenesis: regulators and effectors. Hum Mutat.

[9] Monti P, Ghiorzo P, Menichini P (2017). TP63 mutations are frequent in cutaneous melanoma, support UV etiology, but their role in melanomagenesis is unclear. Oncol Rep.

[10] Kaghad M (1997). Monoallelically expressed gene related to p53 at 1p36, a region frequently deleted in neuroblastoma and other cancers. Cell.

[11] Peters MA, Janer M, Kolb S (2001). Germline mutations in the p73 gene do not predispose to familial prostate-brain cancer. Prostate.

[12] Umar M, Upadhyay R, Khurana R (2012). Role ofp53andp73genes polymorphisms in susceptibility to esophageal cancer: a case control study in a northern Indian population. Mol Biol Rep.

[13] Wang SS, Guo HY, Dong LL (2015). Association between a p73 gene polymorphism and genetic susceptibility to non-small cell lung cancer in the south of China. Asian Pac J Cancer Prev APJCP.

[14] Feng H, Sui L, Du M, et al. Meta-analysis of TP73 polymorphism and cervical cancer. Genet Mol Res 2017;6(1). 10.4238/gmr16016571 28128397

[15] Zhou X, Wu C (2012). Association of p73 G4C14-A4T14 polymorphisms with genetic susceptibilities to breast cancer: a case–control study. Med Oncol.

[16] Ding G, Gu X, Dai Z (2020). Association of genetic polymorphisms in FOXA1 with the progression of genetic susceptibility to gastric cancer. Gastroenterol Res Pract.

[17] Tannapfel A, Schmelzer S, Benicke M, Klimpfinger M, Kohlhaw K, Mössner J (2001). Expression of the p53 homologues p63 and p73 in multiple simultaneous gastric cancer. J Pathol.

[18] Yang ZL, Nie SJ, Zhu H (2013). Association of p53 Arg72Pro polymorphism with bladder cancer: a meta-analysis. Gene.

[19] Niwa Y, Hamajima N, Atsuta Y (2004). Genetic polymorphisms of p73 G4C14-to-A4T14 at exon 2 and p53 Arg72Pro and the risk of cervical cancer in Japanese. Cancer Lett.

[20] Craveiro R, Bravo I, Catarino R, Teixeira AL, Medeiros R (2011). The role of p73 g4c14-to-a4t14 polymorphism in the susceptibility to cervical cancer. DNA Cell Biol.

[21] Feng H, Sui L, Du M, Wang Q. Meta-analysis of tp73 polymorphism and cervical cancer. Genet Mol Res. 2017;16(1). 10.4238/gmr16016571 28128397

[22] Hamajima N, Matsuo K, Suzuki T, Nakamura T, Matsuura A, Hatooka S (2002). No associations of p73 g4c14-to-a4t14 at exon 2 and p53 arg72pro polymorphisms with the risk of digestive tract cancers in Japanese. Cancer Lett.

[23] Lee KE, Hong YS, Kim BG, Kim NY, Lee KM, Kwak JY, Roh MS (2010). p73\r, g4c14 to a4t14 polymorphism is associated with colorectal cancer risk and survival. World J Gastroenterol.

[24] Arfaoui AT, Ben Mahmoud LK, Hmida AB, Khiari M, Mohamed AL, Chaar I (2010). Relationship between p73 polymorphism and the immunohistochemical profile of the full-length (tap73) and nh2-truncated (Δnp73) isoforms in Tunisian patients. Appl Immunohistochem Mol Morphol.

[25] Hu Z, Miao X, Ma H, Tan W, Wang X, Lu D (2005). Dinucleotide polymorphism of p73 gene is associated with a reduced risk of lung cancer in a Chinese population. Int J Cancer.

[26] Li G, Sturgis EM, Wang LE, Chamberlain RM, Wei Q (2004). Association of a p73 exon 2 g4c14-to-a4t14 polymorphism with risk of squamous cell carcinoma of the head and neck. Carcinogenesis.

[27] Choi JE, Kang HG, Chae MH, Kim EJ, Lee WK, Cha SI (2006). No association betweenp73g4c14-to-a4t14 polymorphism and the risk of lung cancer in a Korean population. Biochem Genet.

[28] Liang Z, Weifeng T, Yijun S, Suocheng C, Xu W, Liming W (2014). P21 rs3176352 g>c and p73 rs1801173 c>t polymorphisms are associated with an increased risk of esophageal cancer in a Chinese population. PLoS ONE.

[29] Jemal A, Bray F, Center MM (2011). Global cancerstatistics. CA A Cancer J Clin.

